# Care home staff experiences of delivering diabetes care: a grounded theory study

**DOI:** 10.1186/s12912-026-05062-4

**Published:** 2026-07-19

**Authors:** Stephanie Craig, Tara Anderson, Patrick Stark, Christine Brown Wilson, Claire McEvoy, Fadwa Alhalaiqa, Komal Patel Murali, Yaguang Zheng, Marwa Mamdouh Shaban, Hon Lon Tam, Norbert Buzás, Dragana Milutinović, Berna Dincer, Gary Mitchell

**Affiliations:** 1 School of Nursing and Midwifery, Queen’s University Belfast, Belfast, Northern Ireland; 2 Queen’s University Belfast, Centre for Public Health, Belfast, Northern Ireland; 3 College of Nursing, Qatar University, Doha, Qatar; 4 College of Nursing, New York University, New York, United States of America; 5 Faculty of Nursing, Cairo University, Cairo, Egypt; 6 The Nethersole School of Nursing, The Chinese University of Hong Kong, Hong Kong, SAR China; 7 Faculty of Health Sciences and Social Studies, University of Szeged, Szeged, Hungary; 8 Department of Nursing, Faculty of Medicine, University of Novi Sad, Novi Sad, Serbia; 9 Faculty of Health Sciences, Istanbul Medeniyet University, Istanbul, Türkiye Turkey

**Keywords:** Diabetes, Care homes, Long-term care, Grounded theory, Nursing, Staff perspectives, Multimorbidity, Older people, Qualitative research

## Abstract

**Background:**

Diabetes is highly prevalent among care home residents and is associated with increased clinical complexity due to multimorbidity, frailty, and cognitive impairment. Care home staff play a central role in diabetes management; however, there is limited theoretical understanding of how diabetes care is organised and delivered in long-term care settings. This study aimed to explore care home staff experiences of supporting residents living with diabetes and to develop an empirically grounded explanation of the processes shaping diabetes care in care homes.

**Methods:**

A qualitative study informed by Glaserian grounded theory was conducted. Semi-structured interviews were undertaken with 20 care home staff across 17 care homes in Northern Ireland, including registered nurses, care assistants, home managers, and catering staff. Data collection and analysis proceeded concurrently using constant comparative methods and theoretical sampling. Analysis focused on identifying key processes influencing how diabetes care was prioritised, enacted, and sustained in everyday practice.

**Results:**

Three interrelated categories were identified: Prioritisation, Risk, and Labour. Diabetes education was inconsistently prioritised relative to mandatory training and was unevenly distributed across staff roles. Diabetes-related risk was primarily conceptualised in terms of avoiding acute events, particularly hypoglycaemia, with less attention to longer-term complications or interactions with multimorbidity. Substantial informal and relational labour was undertaken by staff to support residents’ quality of life, negotiate dietary practices, and coordinate care. These categories were integrated through a core category of Misalignment, describing the disconnect between the complexity of diabetes care needs and the organisation of education, roles, and support within care home systems.

**Conclusions:**

Diabetes care in care homes is sustained through adaptation, informal labour, and pragmatic risk management within systems that are not fully aligned to the demands of long-term diabetes care. Addressing this misalignment is essential to strengthening staff capability and informing the development of contextually appropriate educational and service-level interventions for care home settings.

**Clinical trial number:**

Not applicable.

**Supplementary Information:**

The online version contains supplementary material available at https://doi.org/10.1186/s12912-026-05062-4.

## Background

Diabetes mellitus is a complex, long-term condition that continues to pose a major global public health challenge [1]. Type 1 and type 2 diabetes have distinct underlying mechanisms, management requirements and risk profiles. Type 1 diabetes results from autoimmune destruction of pancreatic beta cells and requires lifelong insulin replacement; interruption of insulin can lead to diabetic ketoacidosis, while insulin treatment carries a risk of hypoglycaemia. Type 2 diabetes is characterised by insulin resistance and progressive beta-cell dysfunction and may be managed using lifestyle measures, non-insulin glucose-lowering medicines and/or insulin. Therefore, monitoring requirements and the risk of acute complications vary according to diabetes type and treatment regimen [2–4]. Current estimates suggest that more than 589 million adults worldwide are living with diabetes, with prevalence projected to rise substantially over the coming decades as populations age and rates of multimorbidity increase [5]. In the United Kingdom, diabetes affects approximately 4.6 million people [6], with prevalence disproportionately higher among older adults and those living in socioeconomically disadvantaged contexts [7]. Diabetes is associated with significant morbidity, reduced quality of life, and increased mortality, particularly when glycaemic control is suboptimal or when complications such as cardiovascular disease, neuropathy, renal impairment, and visual loss develop [8, 9].

Diabetes management presents particular challenges in long-term care settings because residents are often older and frailer, live with multimorbidity and cognitive or functional impairment, and may depend on staff for monitoring, medication administration and nutritional support [10, 11]. Estimates consistently suggest that 25% of care home residents live with diabetes, a proportion expected to increase in parallel with demographic change [12]. Unlike community-dwelling adults, residents in care homes are often dependent on staff for blood glucose monitoring, medication administration, nutritional intake, recognition of deterioration, and escalation of care [11]. As such, the quality and safety of diabetes care in care homes is closely linked to staff capability, confidence, and organisational support [13]. This challenge is internationally significant, as long-term care has been identified by the World Health Organization as one of four priority areas for action within the United Nations Decade of Healthy Ageing [14], highlighting the global imperative to strengthen workforce capability and care quality in these settings. However, challenges in diabetes care in care homes are not solely attributable to deficits in individual knowledge or skills, but are embedded within everyday practices, organisational routines, and the wider care system in which staff operate [15].

Managing diabetes in care homes presents distinct clinical and practical challenges [16]. Glycaemic targets often require individualisation to balance long-term risk reduction against the immediate risks of hypoglycaemia, falls, and treatment burden [11]. Cognitive impairment and dementia can limit residents’ ability to recognise or communicate symptoms of hypo- or hyperglycaemia, while irregular appetite, intercurrent illness, and polypharmacy further increase clinical complexity. In this context, care home staff are required to make frequent judgements under uncertainty, often in the absence of immediate access to specialist diabetes support [4]. Diabetes care in care homes should be individualised according to residents’ clinical and functional status, preferences, treatment burden and goals of care, rather than delivered through a uniform approach [11]. However, studies have identified variation in care processes, including monitoring and medication review, across care home settings [17, 18]. What remains poorly understood is how care home staff interpret these competing demands and organise diabetes care in practice, particularly in contexts characterised by uncertainty, time pressure, and limited specialist support [19].

Despite the central role of care home staff in diabetes management, previous research has repeatedly highlighted gaps in training and support. Pre-registration nursing education and post-qualification professional development are commonly oriented towards acute and primary care settings, with limited attention to the specific realities of long-term care and the organisational conditions under which care is delivered [20]. Care support workers (known as care assistants in the UK), who provide the majority of direct care in care homes, often have minimal formal education in diabetes, despite being well placed to observe early changes in residents’ condition [10]. High staff turnover, reliance on agency staff, and constrained access to continuing professional development further limit the sustainability of diabetes expertise within care homes [21].

Additionally, policy and professional guidance increasingly recognise the need to improve diabetes care in care homes. In the UK, the National Advisory Panel on Care Home Diabetes (NAPCHD) was established in response to longstanding concerns about inequity and variability in care, which were further exposed during the COVID-19 pandemic [12]. The advisory panel highlighted the need for improved staff education, more individualised and streamlined approaches to diabetes care, and stronger integration between care homes and external healthcare services. International guidance, including that from the American Diabetes Association and European geriatric medicine societies, similarly advocates for individualised treatment goals, avoidance of overtreatment, and greater attention to staff education in long-term care settings [11, 22]. However, while such guidance outlines *what* should be achieved, there remains limited empirical understanding of *how* diabetes care is delivered in care homes, and how staff navigate the competing demands of safety, workload, resident autonomy, and organisational constraint.

Educational interventions targeting diabetes care in care homes have been explored, but the evidence base remains limited. A recent scoping review identified relatively few empirically evaluated educational interventions, many of which were small-scale, dated, and heterogeneous in content and delivery [23]. Notably, few studies examined how staff incorporated new knowledge into everyday routines, negotiated competing priorities, or adapted guidance within the constraints of care home settings, with most evaluations focusing on knowledge acquisition or discrete practice outcomes rather than the processes underpinning routine care delivery [24–29]. While these interventions generally reported improvements in staff knowledge and confidence, there was limited exploration of how education translated into routine practice, decision-making, or sustained change. Notably, important aspects of diabetes care in older people, such as foot assessment, eye care, and the use of digital or technology-enabled education were inconsistently addressed. These findings highlight the need for a deeper, theory-informed understanding of staff experience to inform the development of acceptable, feasible, and contextually appropriate interventions.

Qualitative research is particularly well suited to addressing this gap, as diabetes care in care homes is shaped not only by clinical guidelines but also by organisational routines, informal knowledge, communication practices, and relational work with residents and families [15, 30]. Grounded theory offers a systematic approach to exploring such complexity, enabling the development of explanatory theory grounded in the accounts of those directly involved in care delivery [31]. In contrast to descriptive qualitative approaches, grounded theory is explicitly concerned with explaining social processes and patterns of action, making it well suited to examining how care is produced and sustained in complex care environments. By focusing on social processes and patterns of action, grounded theory can illuminate how care home staff interpret diabetes, prioritise tasks, respond to uncertainty, and adapt practice within the constraints of their setting. This approach is especially valuable where existing theory is limited and where findings are intended to inform intervention design and implementation. To date, there is a notable absence of grounded theory research examining diabetes care in care homes from the perspective of staff and focused on the processes through which diabetes care is organised and enacted. Understanding how staff experience, organise, and make sense of diabetes care is essential to strengthening workforce capability and improving resident outcomes. Such insight is also critical to informing the co-design of educational and digital interventions that align with care home realities rather than imposing models derived from acute or primary care contexts.

In response to these gaps, this study adopted a grounded theory approach to develop an explanatory account of how diabetes care is understood, organised, and enacted by care home staff supporting older residents. Focusing on staff accounts of routine practice, decision-making, and interaction with wider care systems, the study sought to generate an empirically grounded explanation of the processes shaping diabetes care in care home settings. By elucidating the conditions that influence staff capability, confidence, and care delivery, the findings aim to inform the development of contextually appropriate and sustainable approaches to education and service improvement. As this study did not involve the evaluation of a clinical intervention, trial registration was not applicable.

## Methods

### Aim

The aim of this study was to develop an empirically grounded explanation of how care home staff support residents living with diabetes, and to examine the processes that shape diabetes care within care home settings.

1. To examine care home staff perceptions, experiences, and everyday practices in supporting residents with diabetes.
2. To identify the organisational, educational, and contextual factors that enable or constrain the delivery of diabetes care in care homes.
3. To develop a grounded theory explaining how care home staff navigate diabetes care for older residents within the opportunities and constraints of the care home environment.

### Design

This study adopted a qualitative design informed by Glaserian grounded theory methodology [31] to examine how diabetes care is delivered and supported within care home settings. Grounded theory was selected because there is limited theoretical understanding of how care home staff organise, prioritise and navigate diabetes care for older residents with complex health and social care needs [31–37]. Consistent with Glaserian grounded theory, data collection and analysis were conducted concurrently, using constant comparison to support the development of categories grounded in participants’ accounts [33, 35]. Emerging concepts informed subsequent recruitment and questioning, allowing theoretical sampling to explore developing categories and relationships in greater depth [38–41]. This approach supported the generation of an empirically grounded explanation of the processes shaping diabetes care within the opportunities and constraints of the care home environment.

### Sample

The sample comprised care home staff working in Northern Ireland who were involved in supporting older residents living with diabetes. Participants were recruited from care homes providing long-term care for older people. Staff were eligible if they were aged 18 years or over and had a direct role in diabetes-related care, including monitoring, medication administration, day-to-day care, care planning or food provision. The type of care home in which each participant worked was not recorded; therefore, differences between residential and nursing home contexts could not be examined. Initial purposive sampling sought variation in staff role and level of seniority across the care home workforce [31]. As data collection and analysis progressed, emerging categories informed theoretical sampling, with recruitment extended to participants who could help develop categories relating to organisational context, everyday care and dietary support [33, 36]. Sampling continued until theoretical sufficiency was reached, when further data no longer generated new conceptual insights or altered the properties of the developing categories [35, 39–41]. The final sample included 20 participants from 17 care homes, comprising registered nurses, senior nurses, care assistants, home managers and catering assistants. Table 1 provides an overview of all study participants.

**Table 1:** Demographic details

Participant	Role	Gender	Practice Experience/ Years Qualified (Years)
P1	Care Home Nurse	Female	4
P2	Home Manager	Female	9
P3	Care Assistant	Male	1
P4	Senior Nurse	Female	3
P5	Catering Assistant	Male	2
P6	Home Manager	Female	6
P7	Catering Assistant	Male	4
P8	Care Home Nurse	Female	7
P9	Home Manager	Female	2
P10	Care Assistant	Female	5
P11	Senior Nurse	Female	4
P12	Care Home Nurse	Male	3
P13	Home Manager	Female	11
P14	Care Assistant	Female	1
P15	Catering Assistant	Female	12
P16	Home Manager	Female	5
P17	Care Home Nurse	Female	8
P18	Senior Nurse	Female	6
P19	Care Assistant	Female	2
P20	Care Home Nurse	Female	4

### Recruitment

Participants were recruited through the Royal College of Nursing (RCN) Independent Sector Network in Northern Ireland. This network includes care home staff working across the independent sector, including care home managers, senior nurses and registered nurses. A gatekeeper independent of the research team distributed an invitation email to the network. Staff who wished to participate contacted the research team directly to receive further information and arrange an interview. Detailed organisational characteristics of the participating care homes were not collected. Consistent with grounded theory methodology, recruitment was extended as data collection and analysis progressed, with emerging categories informing subsequent theoretical sampling decisions [31, 33, 36]. Care assistants registered with the Northern Ireland Social Care Council (NISCC) (https://niscc.info/) were subsequently recruited to capture perspectives from staff directly involved in everyday resident care. All participating nurses were registered with the Nursing and Midwifery Council (NMC) (https://www.nmc.org.uk/). During analysis, food provision and everyday nutritional practices became increasingly relevant to the developing categories; therefore, catering assistants were also recruited to further explore diabetes management within the care home environment. Care assistants and catering assistants were not members of the original RCN recruitment network, so snowball sampling was used, whereby existing participants shared study information with eligible colleagues. This was used selectively to access staff groups relevant to the emerging analysis. Interested individuals contacted the research team directly. Written informed consent was obtained electronically at least 48 h before interview and reaffirmed verbally immediately before data collection.

### Data collection

A total of twenty care home staff participated in individual semi-structured interviews, lasting approximately 30–60 min (Supplementary File 1). Interviews were conducted by GM (PhD, Registered Nurse), an experienced qualitative researcher. All interviews were undertaken remotely using Microsoft Teams, which supported participation from staff working variable shifts across geographically dispersed care homes. Participants were drawn from seventeen care homes across Northern Ireland. In three instances, two participants were recruited from the same care home; this occurred during later theoretical sampling and enabled exploration of shared and contrasting perspectives within the same organisational context. Repeat interviews were not conducted. The initial recruitment strategy focused on staff involved in direct resident care. As data collection and analysis progressed, emerging categories highlighted the relevance of wider care home roles, including food provision, and care home managers who had participated supported further recruitment by facilitating contact with relevant staff. The interview topic guide was developed by the Northern Ireland-based members of the research team and reviewed by the wider international academic team. Draft versions were circulated by email and discussed in a research team meeting, during which co-authors commented on clarity, relevance, sequencing and the applicability of questions across different care systems. Where comments reflected country-specific differences in care home organisation, staffing ratios or family involvement, these were considered but not incorporated unless relevant to the Northern Ireland care home context. The guide was also reviewed by the Royal College of Nursing Older People’s Forum steering group, which functioned as a UK clinical expert group. This group, comprised of four registered nurses with direct care home experience, provided feedback on the relevance of the interview questions, recruitment approach and inclusion of staff groups directly involved in day-to-day resident care, including care assistants and care workers. The final topic guide and recruitment approach were agreed by the research team before data collection commenced. Consistent with grounded theory methodology, the topic guide evolved alongside data collection and analysis [31, 34, 36]. As interviews progressed, new questions were introduced and existing questions adapted to explore developing concepts in greater depth. For example, questions were added to examine how staff supported dietary management and blood glucose monitoring in the context of variable appetite and daily routines. Data were collected between April 2025 and May 2025.

### Ethical considerations

Ethical approval for the study was obtained from the Faculty of Health and Life Sciences at Queen’s University Belfast in March 2025 (reference: MHLS 24_167). The research was conducted in accordance with the principles of the Declaration of Helsinki [42]. All participants provided written informed consent prior to participation. Identifiable information was removed during data anonymisation, pseudonyms were assigned, and no data that could reasonably identify individual participants, residents or care homes were retained in the analytic dataset. Interview transcripts and study materials were stored securely on password-protected university systems, with access restricted to the research team. Direct quotations were anonymised before inclusion in the manuscript.

### Data analysis

Data analysis used constant comparative analysis, consistent with Glaserian grounded theory methodology [31, 33, 36]. Interviews were transcribed verbatim and analysed concurrently with data collection. Each transcript was reviewed before subsequent interviews so that emerging insights could inform further questioning and theoretical sampling. Coding was iterative. Initial coding identified meanings, actions and processes within the data, generating provisional conceptual labels. Codes were then compared within and across interviews to examine similarities, differences and patterns. As analysis progressed, related codes were grouped into categories, and relationships between categories were examined and refined through memo-writing, research team discussion and ongoing comparison. The final phase involved integrating categories around a core category that accounted for the relationships between them and provided coherence to the emerging grounded theory [31, 33, 36]. Primary analysis was led by SC and GM, who had qualitative research expertise and approval to access the study data. Transcript-level analysis and coding were undertaken by the UK-based members of the research team with appropriate data access permissions (TA, PS, CMcE, CBW). The wider international academic team did not access raw participant data or undertake coding. Their contribution was consultative, including input on study design, recruitment approach, methodological coherence and discussion of anonymised emerging categories in relation to the developing grounded theory.

### Rigour and reflexivity

Rigour and reflexivity were supported through several strategies. Reporting was informed by the Consolidated Criteria for Reporting Qualitative Research (COREQ) [43] (Supplementary File 2), including attention to study design, data collection, analysis and researcher involvement. As analytical categories developed, selected aspects of the emerging analysis were discussed with three participants to assess resonance with their experiences, while recognising that participants were not positioned as arbiters of theoretical validity [44]. Regular research team discussions were used to examine emerging categories, interpretations and analytic decisions. Memo-writing documented theoretical insights and supported reflexive engagement with the data, while an audit trail recorded key methodological and analytical decisions. Reflexivity was maintained through consideration of how researchers’ clinical, academic and care home experience may have shaped interpretation. Researchers directly involved in data collection and analysis also engaged in peer debriefing with colleagues not involved in the study to consider alternative viewpoints and challenge assumptions.

## Results

Analysis of the interview data generated three categories that described how diabetes care was delivered in care home settings: (1) Prioritisation (2), Risk, and (3) Labour. Each category captured patterned actions and interactions through which staff organised, delivered and adapted diabetes care in everyday practice. Together, these categories informed the core category of Misalignment. Misalignment describes the partial mismatch between residents’ diabetes care needs and the education, role structures, organisational routines and external support available to care home staff. It does not indicate a lack of commitment or poor care; rather, it explains how staff sustained diabetes care through vigilance, adaptation and relational work within systems that were not consistently configured to support holistic, anticipatory diabetes management. Twenty care home staff participated in the study, representing a range of roles and levels of experience. Participant characteristics are presented in Table 1.

### Category 1: Prioritisation of diabetes education

The category of Prioritisation described how diabetes education was positioned within care home settings relative to other training demands and clinical responsibilities. Participants consistently described a hierarchy of learning in which mandatory and regulatory training took precedence, while condition-specific education, including diabetes, was addressed inconsistently and often informally. Although staff recognised diabetes as a common and important condition among residents, it was not routinely prioritised as a focus for structured learning.

Registered nurses commonly drew on knowledge acquired during pre-registration education and early clinical experience. Several described this as providing a broad foundation, particularly in relation to insulin administration and the recognition of acute complications but noted that this knowledge was rarely refreshed or extended once working in care homes. As one nurse explained,

*You’re relying on what you learned years ago at university really. You know the basics*,* insulin*,* hypos*,* that sort of thing*,* but that’s it. There’s nothing new coming in to build on it (P1*,* Care Home Nurse).*

In contrast, care assistants described receiving little or no formal education on diabetes. Most reported learning “*on the job*,” often through observation or instruction from colleagues, rather than through planned training. One care assistant reflected,

*Nobody ever sat us down and said*,* this is diabetes*,* this is what you need to watch for. You just pick it up as you go along*,* or you ask someone if you’re worried (P3*,* Care Assistant).*

This informal learning was often described as pragmatic but incomplete, leaving staff uncertain about the rationale underpinning care decisions. Catering and domestic staff, who played a role in residents’ dietary management, reported even more limited access to diabetes-related education. Those interviewed described relying on general awareness, personal experience, or advice from nursing staff rather than any structured guidance. One catering assistant noted,

*You sort of know sugary stuff isn’t great*,* so you try and be mindful*,* but nobody’s ever given us proper training on diabetes. It’s more common sense than anything else (P5*,* Catering Assistant).*

This lack of formal inclusion reinforced perceptions that diabetes education was not considered a shared responsibility across the care home.

Opportunities for further training were described as uneven. Registered nurses were generally aware of external education provided by healthcare trusts in Northern Ireland, particularly through Clinical Education Centres, and those who had attended described these sessions as useful and relevant. However, access required awareness, time, and organisational support, and was not embedded within routine care home education. As one nurse explained,

*There are courses out there through the [Healthcare] trust*,* and they’re good when you get to them*,* but you have to know about them*,* get the time*,* get the cover. It’s not something that just happens automatically (P6*,* Home Manager).*

Home managers were often described as key facilitators in enabling access to such training, either by identifying opportunities or by cascading information to staff. Where this occurred, participants viewed it positively. One senior care assistant commented,

*If the manager flags something up and says*,* right*,* this is worth going to*,* then you do feel supported. But it depends on who’s in charge and how proactive they are (P14*,* Care Assistant).*

Despite this, participants noted that diabetes education was not mandatory and was rarely delivered in-house, limiting uptake and consistency.

Where diabetes learning did occur within care homes, it was often informal and ad hoc. Some participants described sharing articles, guidance, or online resources among small groups of staff, typically initiated by individuals with a particular interest in the topic. While these efforts were viewed as positive, they were also described as fragmented. As one nurse stated,

*You might read something and pass it on*,* but it’s bits and pieces. There’s no joined-up approach*,* it’s whoever happens to come across something (P12*,* Care Home Nurse).*

The content of diabetes knowledge was also described as narrowly focused. Participants consistently emphasised education related to treatment and the avoidance of acute complications, particularly hypoglycaemia. In contrast, there was limited attention to prevention, psychological impact, or longer-term complications. Several participants framed this in the context of care home admission, where diabetes was perceived as secondary to comfort-focused care. One care assistant explained,

*A lot of the residents are at that stage where it’s about keeping them comfortable. You’re not really thinking prevention anymore*,* and nobody teaches you about that side of things (P10*,* Care Assistant).*

Nevertheless, participants expressed a clear appetite for broader and more detailed education. Areas such as diabetic foot care, neuropathy, and retinopathy were frequently mentioned as topics staff were aware of in principle but felt underprepared to address. A registered nurse reflected,

*You know foot problems are a big issue*,* and eyes as well*,* but we’ve never had proper training on it. You’d like to know what to look for before it becomes a real problem (P18*,* Senior Nurse).*

The category of Prioritisation explains how diabetes education, while widely acknowledged as important, was positioned inconsistently within care home training structures and largely dependent on role, individual initiative, and organisational emphasis.

### Category 2: Managing diabetes-related risk

The category of Risk described how staff identified, interpreted and managed diabetes-related risk in routine care. Participants did not consistently distinguish between type 1 and type 2 diabetes, or specify whether residents were managed by diet, oral glucose-lowering medication, insulin or combined regimens. The category therefore reflects how diabetes-related risk was discussed by staff, rather than assuming that all residents were insulin-dependent or at equivalent risk of hypoglycaemia. In participants’ accounts, risk was most often framed around immediate safety concerns, particularly blood glucose monitoring, medication administration and hypoglycaemia. Longer-term risks, including neuropathy, retinopathy, foot complications and interactions with multimorbidity, were less prominent and were often described in more general terms.

Registered nurses described responsibility for medication administration, including insulin, as a key area of diabetes-related risk. Despite recognising insulin as a high-risk medication, participants did not report medication-related errors within this study. Instead, nurses emphasised vigilance, routine checking, and adherence to care plans as central to safe practice. One nurse explained,

*Insulin is always treated as high risk*,* so there’s checks in place and you’re very aware when you’re giving it. It’s not something you rush*,* and that’s drilled into you (P8*,* Care Home Nurse).*

This sense of caution appeared to contribute to confidence in managing medication-related risk. Experiences of severe acute complications, such as diabetic ketoacidosis or significant hyperglycaemia, were described as rare. Where such events were recalled, they were generally associated with experienced staff and were perceived as being managed effectively without escalation. A senior nurse reflected,

*In all my years*,* I can count on one hand the really serious diabetes incidents. They’re rare*,* and when they do happen*,* they’re picked up early and dealt with (P13*,* Home Manager).*

The relative infrequency of such events contributed to a perception that diabetes-related risk was largely predictable and controllable within the care home environment. Across roles, participants highlighted their familiarity with residents as central to recognising early signs of deterioration. Rather than relying on formal clinical assessment tools, staff described an intuitive awareness of changes from an individual’s usual presentation. This was often framed in relational terms, reflecting the social care context of care homes. One care assistant explained,

*You just know when someone’s not right. It might be they’re quieter*,* not finishing their food*,* just off in themselves. Because it’s their home*,* you notice the small things straight away (P19*,* Care Assistant).*

This collective vigilance extended across nursing, care, and support staff, reinforcing diabetes as a condition that was constantly monitored in everyday practice.

Participants consistently reported that they were aware of which residents had diabetes and which residents required insulin. Insulin use was widely perceived as conferring greater risk and prompted closer monitoring. Blood glucose recording was described as routine, care-planned, and embedded within daily documentation. One nurse commented,

*Blood sugars are always part of the notes*,* it’s there every day. You know who needs checked*,* when*,* and what to do if it’s outside the range (P11*,* Senior Nurse).*

In this sense, diabetes-related safety was described as effectively managed through structured documentation and established routines. However, when discussion shifted beyond acute risk, participants’ accounts became more limited. Longer-term complications of diabetes, such as neuropathy, retinopathy, and foot problems, were less frequently mentioned and were often described in general rather than practical terms. Few participants reported confidence in identifying early signs of these complications, and many were unsure when residents last attended specialist diabetes appointments. One nurse stated,

*You know foot care is important*,* but I couldn’t tell you when most of them last saw podiatry unless I went digging through the notes (P17*,* Care Home Nurse).*

Dietary management represented another area where perceptions of risk were negotiated. Catering and care staff acknowledged that nutritional guidance for diabetes was often balanced against residents’ preferences and the broader issue of malnutrition. Several described a pragmatic approach in which comfort and intake were prioritised over strict glycaemic control. A catering assistant explained,

*If they won’t eat*,* that’s a bigger worry. An extra biscuit or pudding*,* you think*,* well*,* they’re on medication and at least they’re getting something into them (P7*,* Catering Assistant).*

This account illustrated how dietary risk was negotiated when frailty, variable appetite and resident preference intersected with diabetes treatment. The participant did not specify diabetes type or medication regimen, which reflected a broader pattern in which diabetes was often discussed as a single condition rather than by treatment-related risk profile. The presence of multimorbidity strongly shaped how diabetes-related risk was prioritised. Participants consistently emphasised that diabetes was rarely managed in isolation and was often perceived as less urgent than other conditions, such as dementia, heart failure, or chronic obstructive pulmonary disease. These conditions were associated with more visible deterioration, hospital admission, or end-of-life care, and therefore commanded greater attention. One nurse reflected,

*If someone’s got advanced dementia or heart failure*,* that’s where your focus goes. Diabetes is there*,* but it’s not usually what’s going to cause the immediate crisis (P11*,* Senior Nurse).*

Importantly, participants did not make explicit links between diabetes and the progression or exacerbation of other long-term conditions. Diabetes was largely conceptualised as a condition requiring monitoring to prevent acute events, rather than as a contributor to cognitive decline, cardiovascular risk, or overall disease burden. As one care assistant noted,

*You don’t really think of diabetes as affecting the dementia or the heart. You’re more watching it so they don’t go hypo (P14*,* Care Assistant).*

This emphasis on hypoglycaemia reflected the visibility of acute risk in staff accounts, while also indicating that staff did not always articulate distinctions between residents using insulin or other glucose-lowering therapies and those managed without such medicines. The category of Risk captured a pattern in which acute diabetes-related risks were recognised, monitored, and managed effectively within routine care, while longer-term and cumulative risks received less attention. This selective focus on immediacy and safety reflected both the realities of care home practice and the wider context of multimorbidity, within which diabetes was positioned as important but rarely dominant. This emphasis on acute risk management shaped diabetes care as a condition requiring vigilance rather than anticipatory or preventative intervention.

### Category 3: Labour of sustaining diabetes care

The category of Labour reflected the additional work undertaken by staff across roles to support residents to live well with diabetes, much of which was informal, relational, and not formally recognised. This work extended beyond clinical tasks and was embedded within the everyday life of the care home, encompassing emotional support, negotiation, coordination, and connection across people and services.

Nurses and care assistants described diabetes care as closely tied to knowing residents as individuals rather than as diagnoses. Participants emphasised that effective support depended less on technical diabetes knowledge and more on familiarity with residents’ preferences, routines, and responses to illness. One care assistant explained,

*It’s not about knowing diabetes out of a book*,* it’s knowing them. You know what’s normal for that person*,* what they’ll tolerate*,* what they won’t (P14*,* Care Assistant).*

This relational knowledge enabled staff to tailor care in ways that supported quality of life alongside safety. Much of this labour involved managing the psychological and social dimensions of living with diabetes. Participants described supporting residents who felt frustrated by dietary restrictions, anxious about blood glucose monitoring, or distressed by loss of independence. Staff often framed this work as part of helping residents “live normally” within the care home environment. A registered nurse reflected,

*You don’t want diabetes to be the thing that defines their day. It’s about letting them enjoy life as much as they can*,* within reason (P9*,* Home Manager).*

Negotiation emerged as a key feature of this work. Staff described frequent, small-scale decision-making around food and drink, often involving compromise and shared understanding rather than strict enforcement. Examples included discussing sugar in tea, offering alternatives such as sweeteners, or agreeing when an occasional treat was acceptable. One senior care assistant explained,

*You talk it through with them. Maybe it’s*,* ‘How about one sugar instead of two?’ or ‘Could we try the sweetener today?’ It’s about working with them*,* not telling them ‘no’ all the time (P10*,* Care Assistant).*

These negotiations were framed as supporting autonomy and dignity, particularly for residents with capacity. Catering staff played a central role in this labour, despite being less visible within formal care planning. Participants described menus that offered choice while attempting to balance dietary guidance with residents’ preferences. Puddings, biscuits, and snacks remained part of daily routines, but were often adapted to be more diabetes-friendly where possible. A catering assistant noted,

*They still get pudding*,* that’s important to them. We just try and make sensible choices*,* smaller portions*,* different options*,* so it’s not all taken away (P15*,* Catering Assistant).*

This work required creativity and sensitivity, particularly in the context of malnutrition and poor appetite among older residents.

Families were also described as part of the wider labour involved in diabetes care. Staff frequently supported relatives to understand diabetes and its management, particularly where families brought in food or were unaware of dietary implications. One nurse explained,

*Families mean well*,* but they’ll arrive with a big box of chocolates. So you have to have that conversation gently*,* explain why it’s not ideal*,* and suggest alternatives (P20*,* Care Home Nurse).*

Participants also noted that residents sometimes disclosed symptoms or concerns to family members that were not shared with staff, highlighting the importance of communication and partnership. Residents themselves were central to diabetes care, but capacity issues added complexity. Where residents lacked capacity, staff described making decisions in residents’ best interests, often without clear guidance specific to diabetes. This increased the emotional and ethical labour associated with care. A care assistant reflected,

*When they can’t tell you what they’re feeling*,* you’re making judgement calls all the time. You’re thinking*,* what would they have wanted? (P19*,* Care Assistant).*

Beyond the care home, staff described acting as links to external services, including general practitioners, dietitians, and diabetes specialist clinics. Access to these services was widely described as challenging. Participants noted that care homes were often isolated from routine primary care, with General Practitioner (GP) visits limited to urgent issues due to workload pressures. One nurse stated,

*“Getting a GP to come out is very difficult unless it’s critical. You’re often managing things yourself and escalating only when you have no choice” (P4*,* Senior Nurse).*

While blood tests could be arranged, much diabetes care was described as being managed “at a distance,” away from the bedside. Dietetic input was described as particularly difficult to access, with long waiting times and limited availability. Participants reported little engagement with diabetes specialist services and no links with regional or voluntary diabetes organisations. As one home manager noted,

*You’re very much on your own as a care home. We’re independent*,* so you don’t have the same built-in supports. The staff end up holding it all together (P2*,* Home Manager).*

Exercise and physical activity were rarely discussed in relation to diabetes management. While participants acknowledged the importance of mobility, activity was generally framed around wellbeing or fall prevention rather than glycaemic control. Structured exercise as a component of diabetes care was largely absent from accounts, reflecting both practical constraints and limited emphasis within training.

The category of Labour captured the extensive, interconnected work undertaken by care home staff to sustain diabetes care across clinical, social, and organisational boundaries. This labour was essential to residents’ day-to-day experience of living with diabetes, yet remained largely informal, relational, and unsupported within wider systems of care.

### Core category: Misalignment

The core category of Misalignment integrates the categories of Prioritisation, Risk and Labour to explain the central process underpinning diabetes care delivery in care home settings. Misalignment was evident across all three categories. It was reflected in the inconsistent prioritisation of diabetes education, the emphasis on immediate safety over longer-term diabetes-related risk, and the substantial informal work undertaken by staff to support residents’ quality of life. Staff were not passive within this misalignment. Participants consistently described attentive, person-centred care, including recognising changes in residents’ usual presentation, negotiating dietary choices, supporting autonomy and coordinating with families and external services. However, this work often relied on individual judgement, experience and local adaptation rather than systematically aligned education, role preparation or service support.

Misalignment was first apparent in how diabetes education was positioned relative to other training priorities. While staff across roles recognised diabetes as a common and important condition among residents, education relating to diabetes was inconsistently embedded and often secondary to mandatory or regulatory training. This resulted in a gap between what staff were expected to manage in practice and the preparation they received to do so. As one nurse reflected,

*You’re dealing with diabetes every day*,* but the training doesn’t really match that reality. It’s there in bits*,* but it’s not joined up (P11*,* Senior Nurse).*

This lack of alignment between educational provision and clinical demand shaped how diabetes care was subsequently understood and delivered.

The core category of misalignment was further reinforced through the way diabetes-related risk was conceptualised and managed. Acute risks, particularly hypoglycaemia, were highly visible and actively monitored through care plans, documentation, and routine practice. Staff demonstrated confidence in recognising and responding to immediate deviations from residents’ usual presentation, often drawing on relational knowledge and familiarity rather than formal assessment frameworks. However, longer-term and cumulative risks associated with diabetes were less prominent in participants’ accounts. Diabetes was rarely discussed in relation to its interaction with other long-term conditions, such as dementia or heart failure, despite staff caring for residents with complex multimorbidity. This selective focus reflected a misalignment between the chronic, progressive nature of diabetes and a system oriented toward managing immediate safety concerns. An additional dimension of misalignment concerned the limited differentiation in some staff accounts between diabetes type, treatment regimen and risk profile, despite these distinctions being clinically important for day-to-day management.

Misalignment was also evident in how responsibility for diabetes care was distributed across roles. The category of Labour illustrated that much of the work required to support residents with diabetes occurred outside formal clinical tasks and was embedded within everyday interactions, negotiations, and coordination. Staff described undertaking significant emotional, social, and relational work to support residents’ quality of life, often compensating for gaps in formal guidance or external support. One care assistant explained,

*A lot of what we do isn’t written anywhere. It’s just knowing them*,* talking things through*,* keeping them settled. That’s the bit people don’t see (P14*,* Care Assistant).*

This labour was essential to sustaining diabetes care but remained largely invisible within organisational structures.

The misalignment extended beyond the care home itself and into relationships with external health services. Participants described limited access to specialist diabetes support, infrequent GP involvement, and challenges in securing dietetic input. Care homes were described as operating at a distance from primary and specialist care, with staff acting as intermediaries to coordinate services that were often difficult to access. As one nurse noted,

*You’re kind of holding everything together from inside the home*,* but the services are all outside it (P1*,* Care Home Nurse).*

This positioning reinforced care homes’ relative isolation and placed additional responsibility on staff to manage complex care with limited external integration. Importantly, misalignment did not equate to an absence of care or commitment. Participants consistently described providing attentive, person-centred support and expressed pride in their ability to recognise change, maintain safety, and support residents’ wellbeing. However, this care was often delivered despite, rather than because of, existing systems. The alignment between residents’ needs, staff expertise, organisational priorities, and external services was partial and fragile, relying heavily on individual effort, experience, and local workarounds.

Across the three categories, misalignment functioned as a dynamic and ongoing condition rather than a static deficit. Staff continuously negotiated priorities, balanced risk and quality of life, and coordinated care across roles and boundaries. One participant summarised this tension succinctly:

*You’re always trying to do the right thing*,* but the system doesn’t always make it easy to do it well (P10*,* Care Assistant).*

This statement captures the essence of misalignment as the core phenomenon underpinning diabetes care in care home settings. Misalignment provides an explanatory framework for understanding how diabetes care is delivered within care homes. It highlights the gap between the complexity of diabetes as a long-term condition and the organisational, educational, and service structures intended to support its management. By integrating the categories of Prioritisation, Risk, and Labour, misalignment offers a coherent account of why diabetes care is sustained through adaptation, negotiation, and relational work, rather than through systematically aligned support.

**Fig. 1:**
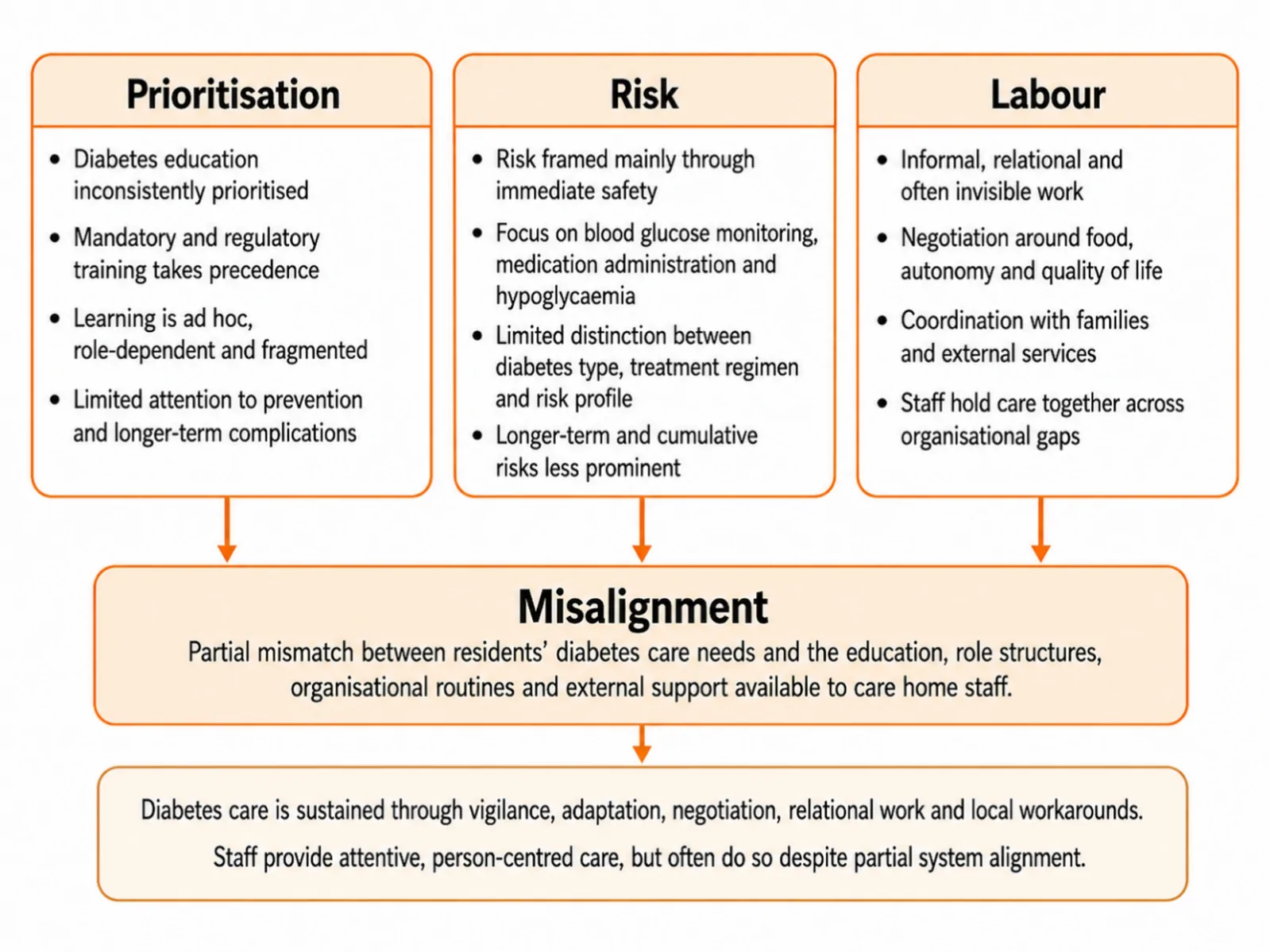
Grounded theory model of misalignment in diabetes care in care homes

The relationships between Prioritisation, Risk, Labour and the core category of Misalignment are presented in Fig. 1. The model illustrates how diabetes care in care homes was shaped by partial mismatch between residents’ needs and the educational, organisational and service structures available to staff, with care sustained through ongoing adaptation, vigilance and relational work.

## Discussion

This grounded theory study provides an explanatory account of how diabetes care is delivered and supported within care home settings. The core category of misalignment explains the partial and fragile fit between residents’ diabetes care needs and the education, role structures, organisational routines and external support available to care home staff. Misalignment, as used in this study, does not indicate poor care or lack of commitment. Rather, it captures how staff work creatively, relationally and ethically to maintain safe, person-centred care within systems that are not consistently configured to support the longitudinal, anticipatory and multidisciplinary nature of diabetes management. In doing so, the study extends descriptive research on diabetes care in long-term care by identifying the organisational and relational processes through which diabetes care is sustained in everyday practice [11, 45–48].

The first dimension of misalignment concerned the prioritisation of diabetes education. Participants recognised diabetes as common and clinically important, but diabetes-related learning was not consistently embedded within formal training structures. Instead, education was described as uneven, role-dependent and frequently acquired through experience or informal workplace learning. This aligns with wider evidence that care home education is often shaped by regulatory and mandatory training requirements rather than the complexity of residents’ long-term conditions [49–51]. Similar patterns have been reported internationally, where condition-specific education is often secondary to compliance-focused training, contributing to fragmented knowledge across staff groups [52, 53]. The present study adds to this evidence by showing how the positioning of diabetes education shapes practice, in that, staff were expected to contribute to diabetes care, but preparation for this work was not consistently shared across roles. Care assistants, managers and catering staff also contributed to residents’ day-to-day care, including monitoring change, supporting routines, negotiating food choices and escalating concerns. However, education and guidance remained largely oriented towards registered nursing roles. This finding resonates with evidence that healthcare assistants provide much direct chronic illness care in long-term care settings while often remaining peripheral to formal education and decision-making structures [54, 55]. In diabetes care, where food provision, daily observation and recognition of deterioration are central to safe support, limited inclusion of non-registered staff in structured education represents a key source of misalignment between care demands and organisational support [56].

A second dimension of misalignment concerned how diabetes-related risk was understood and managed. Participants’ accounts were dominated by immediate and visible risks, particularly hypoglycaemia, blood glucose monitoring and medication administration. This focus is clinically understandable, given the vulnerability of older adults to hypoglycaemia and its association with falls, cognitive impairment and hospitalisation [57, 58]. It also reflects contemporary guidance that emphasises relaxed glycaemic targets and avoidance of overtreatment in frail older adults or those with limited life expectancy [11, 16, 59]. However, longer-term and cumulative risks, including neuropathy, foot disease, vascular complications and interactions with multimorbidity, were less visible in everyday accounts [60]. Diabetes was rarely described as contributing to wider trajectories of cognitive decline, cardiovascular disease, functional impairment or mortality, despite established epidemiological evidence linking diabetes with these outcomes in later life [61, 62]. This selective focus reflects a pragmatic orientation to immediate safety within pressured care environments, but it may also contribute to the under-recognition of preventable diabetes-related complications [63, 64]. This study also identified limited distinction in some staff accounts between diabetes type, treatment regimen and risk profile. This finding should not be interpreted as evidence that staff assumed all residents were insulin dependent. Rather, it demonstrates how diabetes was often discussed as a single care issue, despite important clinical differences between residents managed by diet, oral glucose-lowering medicines, insulin or combined regimens. This further illustrates misalignment between the complexity of diabetes management and the educational, documentation and decision-support structures available in care homes.

The third dimension of misalignment concerned the labour required to sustain diabetes care. Participants described extensive relational, emotional and coordinative work, including supporting residents’ autonomy, responding to distress, negotiating food choices, communicating with families and coordinating external services. Much of this work was not formally recognised within role descriptions, training, documentation or resource allocation. These findings align with sociological accounts of care work as relational, morally situated and often rendered invisible within organisational systems [65–68]. In this study, such labour was not peripheral to diabetes care; it was central to how care was sustained. Food and dietary practices were particularly illustrative. Staff did not describe rigid enforcement of dietary restriction, but ongoing negotiation between glycaemic control, nutritional intake, resident preference, frailty and quality of life. This reflects a broader shift in gerontological diabetes care towards individualised and pragmatic approaches that prioritise comfort, dignity and quality of life [11, 69]. It also resonates with Mol’s “logic of care”, where good care is achieved through responsiveness, practical judgement and continual adjustment rather than strict adherence to predefined rules [70]. In care homes, diabetes management therefore becomes a relational and ethical practice as well as a clinical one. Misalignment also extended beyond the care home. Participants described limited access to specialist diabetes support, dietetics, podiatry and general practice, with staff often acting as intermediaries between residents, families and external services. This reflects wider evidence that care homes may experience fragmented relationships with external healthcare systems, placing additional coordination demands on staff and increasing reliance on informal advocacy [71–73]. Such fragmentation shifts responsibility for system navigation onto frontline staff and embeds diabetes care within invisible labour rather than formal support structures.

Overall, the categories of prioritisation, risk and labour explain why diabetes care in care homes is sustained through adaptation, vigilance, negotiation and relational work. Existing studies and evaluations of diabetes education in care homes have often focused on knowledge deficits, educational need or discrete practice outcomes [23–26, 74–76]. This study extends that literature by showing that diabetes care is not limited by what individual staff know, but by how education, responsibility, risk and external support are organised around them. Future educational and digital interventions should therefore be co-designed with the full range of staff involved in residents’ day-to-day diabetes care. Interventions should support collective capability, recognise the relational labour already undertaken by staff, and strengthen links between care homes and external diabetes services. Addressing misalignment requires moving beyond reliance on individual initiative and invisible work towards more sustainable, system-supported diabetes care in care homes.

### Strengths and limitations

A key strength of this study is that it generated an empirically grounded explanation of how diabetes care is delivered and supported within care home settings. The inclusion of registered nurses, care assistants, home managers and catering staff enabled the analysis to capture the distributed nature of diabetes care and the clinical, relational and organisational work involved in supporting residents. Rigour was supported through concurrent data collection and analysis, constant comparison, theoretical sampling, COREQ-informed reporting [43], participant resonance discussions, reflexive team discussion and an audit trail. The international academic team contributed to study design, topic guide development, recruitment planning and discussion of anonymised emerging categories; however, transcript-level analysis was undertaken by UK-based researchers with appropriate data access permissions. Some limitations should be acknowledged. The study was conducted in one geographical region, which may limit transferability to care home systems with different organisational, regulatory or workforce contexts. The type of care home in which participants worked was not recorded; therefore, differences between residential and nursing home settings, including residents’ level of dependency or capacity to self-manage diabetes, could not be examined. Detailed organisational characteristics of participating homes were also not collected, so differences according to provider context or funding arrangements could not be explored. The study focused on staff perspectives only; resident and family experiences remain important areas for future research. Participation was voluntary, so staff with a particular interest in diabetes care may have been more likely to take part. Finally, the findings are based on interview accounts rather than direct observation, which may limit insight into how diabetes care is delivered in situ. Despite these limitations, the study provides a theory-informed account of diabetes care in care homes that can inform future educational and service-level interventions.

## Conclusion

This study offers a grounded explanation of how diabetes care is organised and delivered in care home settings. The findings highlight a partial misalignment between the complexity of diabetes care and the ways in which education, roles, and everyday practices are structured within care homes. Diabetes management was largely shaped by competing priorities, a focus on immediate clinical risk, and substantial informal labour across staff roles. This misalignment did not reflect poor care or lack of commitment; rather, staff sustained diabetes care through vigilance, adaptation, negotiation and person-centred support. While staff demonstrated strong commitment to resident safety, diabetes education and support were inconsistently embedded. Addressing this misalignment is essential to strengthening diabetes care and informing the development of contextually appropriate educational and service-level interventions that recognise distributed responsibility and support more systematic, sustainable care.

## Supplementary Information

Below is the link to the electronic supplementary material.

Supplementary Material 1

Supplementary Material 2

## Data Availability

The datasets used and/or analysed during the current study are available from the corresponding author on reasonable request.
