# Supplementary material for: Care home staff experiences of delivering diabetes care: a grounded theory study
Source: BMC Nurs. 2026 Jul 19;25:1100. doi: 10.1186/s12912-026-05062-4 (PMC13757128; doi:10.1186/s12912-026-05062-4)
Supplement: Supplementary file 1 [file 12912_2026_5062_MOESM1_ESM.docx]

**Re: Nurses’ attitudes towards diabetes in older people living in care homes**

**Interview Protocol**

The interviewer will introduce him/herself and reiterate the purpose of the interview, i.e. to explore nurses’ attitudes towards diabetes healthcare provision for older adults living in care homes, and the barriers they experience providing this type of service. The interviewer will reiterate that the interview will be audio recorded (via MS Teams) and transcribed for research purposes, but transcripts will be anonymised i.e. will not contain participant names. In addition, it will be made clear to participants that they can inform the facilitator if there are any statements they do not wish to be transcribed at the end of the session. The interviewer will explain to the participants that they are free to stop the interview at any stage if they feel uncomfortable with any of the discussions or do not wish to participate further.

The interviewer will ask if participants have any questions before starting the discussion.

**Interviewer guide:**

Care home nursing healthcare provision

- What role to you have in relation to providing or supporting the diabetes healthcare of older patients living in a care home?
- To what extent do patients have their diabetes condition regularly assessed by care home nurses?
- Is there a particular profile of patient that requires higher levels of diabetes care in the care home?
- Other than care home nurses, what other people have a key role in the provision of diabetes care to these patients (e.g., family members, auxiliary, GP, other specialists).
- How much input does your care home nursing service have with diabetes specialist colleagues (e.g., diabetes specialist nurses)?
- What sort of education or training is provided for care home nurses about diabetes care of older patients and what are your thoughts on this? (e.g. is it mandatory training, does it meet your professional needs?)

Views and perceptions of diabetes care service for older people living in care homes

- How important do you feel it is for care home nurses to be involved in the diabetes care of residents?
- What do you believe are the main barriers for care home nurses in providing diabetes care to people living in care homes? (e.g. COVID-19, financial and time constraints, lack of equipment, lack of knowledge, safety, complex needs etc.)
- What helps you as a care home nurse to ensure optimum practice in relation to providing diabetes care to your residents.
- How do you think we can improve provision of diabetes care for older people living in care home settings?

*Conclusion:*

- Do you have anything else you would like to add that we haven’t already discussed?
- Thank you for your contribution.
- Ask the participant if there are any particular statements that they do not wish to be transcribed.
